# Wastewater analysis for new psychoactive substances and cocaine and cannabis in a Northern Ireland Prison

**DOI:** 10.1038/s41598-023-44453-4

**Published:** 2023-10-30

**Authors:** Bethan Davies, Richard Paul, David Osselton

**Affiliations:** 1 Bournemouth University, Christchurch House C205, Talbot Campus, Fern Barrow, Poole, BH12 5BB UK; 2 Bournemouth University, Christchurch House C239, Talbot Campus, Fern Barrow, Poole, BH12 5BB UK

**Keywords:** Biochemistry, Environmental sciences, Chemistry

## Abstract

The global drug market has been significantly impacted by the emergence of new psychoactive substances, leading to challenges in creating effective legislative controls and their use within recreational drug consumption. This research explores the prevalence of new psychoactive substances and non-medicinal and medicinal compounds within a prison facility in Northern Ireland. Wastewater samples collected from seven different manholes within the prison were analysed for 37 target compounds including the two most found illicit substances: benzoylecgonine (primary metabolite of cocaine) and cannabis. Using solid phase extraction with Oasis HLB and liquid-chromatography-time-of-flight-mass spectrometry across a gradient of 9 min, our analysis revealed that benzoylecgonine was the sole compound consistently present in all collected samples. Following this finding, our target compound selection was broadened to encompass medicinal compounds and employing qualitative analysis we re-evaluated the samples and discovered the presence of buprenorphine, benzodiazepines, methadone, morphine, and codeine. Finally, the study explored the application of enzymatic beta-glucuronidase hydrolysis to the samples. This final phase yielded significant findings, indicating the presence of codeine and nordiazepam at higher peak intensities, thereby shedding light on the potential implications of this enzymatic process.

## Introduction

In recent years, new psychoactive substances (NPS) have established themselves within society as a distinct category of substances. NPS is described as a heterogeneous group of substances associated with several health and social harms on an individual and societal level^1^. In 2020 alone, 7 tonnes of NPS were seized across Europe and by the end of 2021, 880 NPS were being monitored by the European Monitoring Centre for Drugs and Drug Addiction (EMCDDA)^2^ Across the globe, numerous legislations (Prisons Act 2021) and practices have been brought into effect to deal with the harms associated with the usage of NPS and to reduce their supply and production^2–5^. The overall use of NPS within the UK has remained low in comparison to other more traditional illicit substances (i.e., cocaine, cannabis) but the greatest concern is an increase in use and harm within high-risk groups i.e., homelessness, prison, or even high-poverty areas^6^. The Office of National Statistics published in August 2022, that the number of deaths in 2021 related to drug abuse/poisoning including NPS has increased since 2020 from 4,561 to 4,859 therefore, the number of deaths has nearly doubled in the last 10 years since 2012, up from 2,572 deaths^7^. 258 deaths in 2021 within that data set were deaths due to NPS specifically, an increase from 137 deaths in 2020^8,9^.

In recent years, wastewater analysis has become a popular tool for determining illicit substances and NPS at a population level, providing insight into society's drug abuse trends^10^. Wastewater analysis is gaining popularity and can provide useful information. The analysis can be challenging because it involves the analysis of influent wastewater collected before any treatment, to provide a population-wide estimate of human consumption within the captured area. This has previously been successfully conducted in Australia, China, and Europe^8–10^.

This study aimed to evaluate the potential presence of thirty-seven target compounds, a combination of NPS, non-medicinal and medicinal analytes in the wastewater from sewage manholes located at a Northern Ireland Prison. The study provides a validated LC-ToF–MS method using a data-independent acquisition form of quantification. The results presented in this study can be used to provide avenues for future wastewater studies. Ethical approval for this study was obtained by Bournemouth University Ethics Committee (Ref ID: 25313).

## Materials and methodology

### Northern Ireland prison

A Northern Ireland Prison sewage system was monitored over a day in 2022. The samples were collected from 7 different sewage manholes. Each manhole corresponds to a particular in-mates ‘house’ on the prison grounds. Samples were labelled 1–7 to maintain anonymity between the in-mate houses. All samples were spot collections using 250 mL polypropylene bottles. The prison is a maximum-security facility with an inmate capacity of 1940 (as of 2020) with approximately 300 inmates per house^11^. Permission for water sampling at the prison was granted by the Governors and Senior Officers. The analyte selection for this study was based on commercial tender requirements, specific requests by the Prison and recurring submissions to projects such as WEDINOS.

### Sample collection

Single 250 mL wastewater samples were collected from 7 different manholes located on the prison grounds. Each manhole was secured before collection. A polypropylene bottle was attached to a sampling rod purchased from Merck (Darmstadt, Germany) the rod was lowered into the manhole and a 250 mL spot sample was collected. The data and time of collection were noted on each bottle. Each manhole corresponds to a particular in-mates ‘house’ on the prison grounds. Samples were labelled 1–7 to maintain anonymity between the in-mate houses. Samples were stored in the freezer for 2 weeks and given 1 day to defrost before analysis. NPS has been shown to be stable in frozen wastewater for at least this period of time^12–14^. All samples were collected from manholes with free-flowing wastewater all of which are the main pipeline directing away from each inmate housing. During the time of collection, a minimum of 60% of the inmate population was confined to their living quarters.

### Analytical materials

All analytes present and the corresponding deuterated internal standards were purchased from either Chiron (Norway) or Merck (Darmstadt, Germany) at concentrations of 1 mg/mL or 100 ug/mL in methanol (MeOH) or acetonitrile (AcN). Dilutions and working standard mixtures with concentrations ranging between 20 and 1000 ng/L were further prepared using HPLC grade MeOH. HPLC grade AcN, MeOH and formic acid were purchased from Rathburn Chemicals (Walkerburn, UK) whereas the ammonium acetate was purchased from Merck (Darmstedt, Germany). Ultrapure water was obtained by purifying tap water in an ELIX Millipore water purifier obtained from Millipore (Darmstedt, Germany). Oasis HLB (500 mg, 6 cc) SPE cartridges were purchased from Waters (New Bedford, MA, USA).

### Sample preparation and solid phase extraction

Samples were kept frozen until analysis and given 1 day to defrost in a fridge ranging between 3 and 8 °C.

All samples were subjected to solid-phase extraction (SPE) and the same procedure was used for all investigated analytes. The SPE procedure is an adaptation of the protocol conducted by van Nuijs et al.^15^. The universal, polymeric reverse-phase SPE cartridges Waters HLB 500 mg, 6 cc (New Bedford, MA, USA) were used for this method. In detail, samples (25 mL) were spiked with 100 µL of a mixed internal standard solution at 50 ng/mL. The SPE cartridges were conditioned with 6 mL methanol and 6 mL deionised water. The samples were then passed through the cartridges under vacuum at a 5 mL/min rate. Cartridges were then washed with 3 mL of deionised water followed by vacuum drying for 5 min. Elution occurred with 4 mL of methanol followed by an additional 4 mL of methanol. The eluents were dried using a sample concentrator attached to a heating block set at 55 °C. Samples were then reconstituted using 100 µL of HPLC grade acetonitrile followed by 100 µL of 5 mM ammonium acetate. All samples were then transferred to a 96-deep well plate for analysis.

### Instrumentation and method validation

37 compounds were targeted using an AB Sciex 5600 + liquid chromatography time-of-flight mass spectrometry (LC-ToF–MS) coupled with an electrospray ionisation (ESI) source (Table S1). Chromatographic separation was performed using a YMC-Triart Phenyl 450 bar column (12 nm, 5 µm, 100 × 3 mm) (Crawford Scientific, UK). Separation was carried out on a gradient method over 9 min. 5 mM ammonium acetate 0.2% formic acid (A) and methanol. (B) was used for the mobile phases.

Quantitative analysis was performed using ToF–MS utilising Sequential Window Acquisition of all THeoretical fragment-ion (SWATH) acquisition. The SWATH windows range between 175 m/z for the first window, ending at 505 m/z, all windows were present in positive ionisation mode. Every compound was quantified within the monoisotopic mass corresponding to a particular SWATH window.

Recovery was investigated using a combined methanolic standard with a concentration of 50 ng/mL, spiked into wastewater. Due to the high number of target analytes with multiple physiochemical properties, previous studies suggest the optimum SPE cartridge use is the Oasis HLB^16–19^. Oasis HLB provided an average recovery of 46% for all 37 compounds.

Linearity consisted of six calibration points at the following concentrations 20, 50, 100, 250, 500 and 1000 ng/L. The calibration curve was used for the quantification of grab wastewater samples from prison manholes. 10 internal standards were selected to cover the whole acquisition method due to the unavailability of internal standards for all 37 compounds, commercially. A 6-point calibration (R^2^ > 0.99) from 20 ng/L to 1000 ng/L was achieved for all 37 compounds. LOD was calculated between 4 and 20 ng/L for all analytes in spiked wastewater. LOQ was deemed to be the lowest calibrator level at 20 ng/L.

The intra- and inter-day accuracy and precision of the method were assessed using three different concentration levels across the linear range, situated at the lower end (80 ng/L), mid-range (300 ng/L) and top end (800 ng/L) in spiked wastewater. Inter-day and intra-day mean accuracy was calculated between 77 and 100%, the intra-day precision ranged between 8 and 20%, and the inter-day precision ranged between 7 and 30%, all using ANOVA as the statistical tool.

## Results and discussion

The results of the measured concentration for each analyte and metabolite for each sample will be discussed in the following sections.

### Illicit drugs and metabolites concentration

Table 1 summarises the measured concentrations for each target analyte. From a total of 37 compounds, only benzoylecgonine was detected in the wastewater samples. The highest benzoylecgonine concentration was detected at 4000 ng/L from prison manhole 7. There is no significant difference between the results from each prison manhole. This may create an impression that there is a stable cocaine issue within this prison, especially within the inmate houses.

**Table 1:** Results from spot sewerage collected from 7 different manholes on the prison site.

Analyte	Sample number (Manhole number)						
	1	2	3	4	5	6	7
5C-NBOMe	ND	ND	ND	ND	ND	ND	ND
25I-NBOMe	ND	ND	ND	ND	ND	ND	ND
2C-B	ND	ND	ND	ND	ND	ND	ND
2-OXO-LSD	ND	ND	ND	ND	ND	ND	ND
4-Methylethcathinone	ND	ND	ND	ND	ND	ND	ND
5F-AB-PINACA	ND	ND	ND	ND	ND	ND	ND
5F-APICA	ND	ND	ND	ND	ND	ND	ND
5F-APINACA	ND	ND	ND	ND	ND	ND	ND
5F-MDMB-PINACA	ND	ND	ND	ND	ND	ND	ND
5F-PB-22	ND	ND	ND	ND	ND	ND	ND
5-MeO-DALT	ND	ND	ND	ND	ND	ND	ND
AB-FUBINACA	ND	ND	ND	ND	ND	ND	ND
AB-PINACA	ND	ND	ND	ND	ND	ND	ND
AB-PINACA metabolite	ND	ND	ND	ND	ND	ND	ND
Alprazolam	ND	ND	ND	ND	ND	ND	ND
AM2201 4-Hydroxypentyl	ND	ND	ND	ND	ND	ND	ND
APICA 4-hydroxypentyl	ND	ND	ND	ND	ND	ND	ND
APINACA 4-hydroxypentyl	ND	ND	ND	ND	ND	ND	ND
APINACA 5-hydroxypentyl	ND	ND	ND	ND	ND	ND	ND
Benzoylecgonine	3.0	3.0	2.0	1.5	2.2	1.2	4.0
Benzyl piperazine	ND	ND	ND	ND	ND	ND	ND
Etizolam	ND	ND	ND	ND	ND	ND	ND
Fentanyl	ND	ND	ND	ND	ND	ND	ND
JWH-018 pentanoic acid	ND	ND	ND	ND	ND	ND	ND
LSD	ND	ND	ND	ND	ND	ND	ND
MDMB-CHMICA	ND	ND	ND	ND	ND	ND	ND
MDPV	ND	ND	ND	ND	ND	ND	ND
Mephedrone	ND	ND	ND	ND	ND	ND	ND
Methoxetamine	ND	ND	ND	ND	ND	ND	ND
Methylone	ND	ND	ND	ND	ND	ND	ND
Norfentanyl	ND	ND	ND	ND	ND	ND	ND
PB-22 carboxyindole	ND	ND	ND	ND	ND	ND	ND
TFMPP	ND	ND	ND	ND	ND	ND	ND
THC-COOH	ND	ND	ND	ND	ND	ND	ND
UR-144 4-Hydroxypentyl	ND	ND	ND	ND	ND	ND	ND
UR-144 5-Hydroxypentyl	ND	ND	ND	ND	ND	ND	ND
UR-144 COOH	ND	ND	ND	ND	ND	ND	ND

*N.D* not detected.

There have been very few wastewater studies within a prison setting, which can help with the determination of illicit substances and medication. A study conducted by Postigo et al. in 2011 demonstrated the presence of cocaine and 11-nor-9-carboxy-THC (THCC) along with other medicinal compounds such as alprazolam, methadone, and morphine^20^. Two other studies using wastewater in prison have been conducted in Australia and the USA^21,22^. Both studies concluded that benzoylecgonine was not detected above LOD for the 24-h crude samples collected at the prison for the study by Brewer et al. 2016 and van Dyken et al. 2016. The reasoning behind this is that the authors believe that cocaine is less likely to be used within a prison setting compared to the abuse of medicinal substances^21,22^. Therefore, there is a variation between what substances are abused within prison settings based on country. A sewage study conducted in France, which looked at a prison setting for illicit substance use, stated that psychoactive substance use among inmates is poorly documented in France and has been based on qualitative results from inmate surveys^23^.

As our study was a targeted analysis rather than a non-targeted analysis, the known dynamic tendencies of NPS compounds and the amount of time taken to develop and validate a targeted analysis method, could mean that NPS compounds possibly present within the prison could have already evolved. NPS compounds are normally present at significantly lower levels than more traditional compounds (e.g. cocaine), and the method by which substances are entered into prison settings may have an impact on the amount of substance consumed. One method employed to bring NPS into prison settings that has been studied recently is the impregnation of letters^24^. Ford and Berg^24^ demonstrated that substances such as cocaine, AB-PINACA, AB-FUBINACA and MDMB-CHMICA were being smuggled into UK prisons via this method; all these compounds were included in our study.

A more recent study explored the different ways drugs are smuggled into prisons, the trends, and drug usage on a global scale^25^. This study discussed the most common ways that illicit substances may enter a prison setting. The main entry pathways are prisoners themselves, especially prisoners who are on work release, general release or through visitors. Mail, as seen by Giorgetti et al. 2022, is still an ongoing issue as recently, a synthetic cannabinoid was detected in a letter sent to a German prisoner^26^. It is instances like this that have changed drug strategy within prisons. HMP Liverpool, UK now photocopies any letters to prisoners before the inmate receives them^27^. Instead of letters, the UK has reported that drugs have been laced within underwear garments and socks^28^. Staff are the third mechanism for drugs entering prisons. Corrupt staff are a great cause of concern worldwide for dealing drugs and other contraband^25^. As technology has advanced, the methods by which drugs are entering prisons have advanced too. Drones are now being used to drop packages over the perimeter of the prison including prisons in Germany, Poland, the USA, and the UK^29^. There is anti-drone technology available to prevent drones from entering the prison perimeter but many countries including the UK and the USA have national legislation that prohibits any interferences with a drone’s radio signal or attempts to capture/shoot down a drone before entering the prison’s perimeter leaving very little response time following any drone sightings^29,30^.

### Qualitative analysis of illicit drugs and metabolites

As no NPS substances were detected in the main pilot study, and the targeted method was restricted to the compounds validated within the method, a qualitative analysis was carried out by re-processing the already acquired manhole crude samples using Sciex PeakView software to determine if any medicinal compounds are present but were not included in the targeted method.

These compounds were chosen because these analytes are the most common medicines prescribed in the UK^31^. The results were determined by looking at each compound's peak height and area of the monoisotopic mass. As this is a qualitative approach and exact concentration cannot be determined, only a determination of whether a compound is present or not present can be reported. All 7 of the manhole, spot, and crude samples were re-processed for the compounds listed in Table 2. Table 3 provides a breakdown of the results.

**Table 2:** List of target analytes for qualitative analysis and their corresponding mass.

Analyte	Mass
Methadone	310.2
Buprenorphine	468.3
Oxazepam	287.0
Morphine	286.1
Codeine	300.1
Nordiazepam	271.0

**Table 3:** Results from the qualitative analysis of 7 crude samples using PeakView.

Sample number	Analytes present
1	Buprenorphine, codeine, methadone
2	None detected
3	None detected
4	Codeine
5	None detected
6	Oxazepam, codeine, morphine, nordiazepam
7	None detected

The results provide evidence that there is a presence of medicinal substances within the wastewater samples from the prison. This is not unusual for a prison setting as it’s well known that prisoners have access to health care whilst serving their sentences. The UK has an ageing population which can contribute to the increasing number of medicinal substances being consumed. This is no different within a prison setting. The number of prisoners over the age of 50 has grown by nearly 200% within the last 15 years^32^. Diazepam was not chosen as a target analyte as diazepam is not commonly seen in urine and based on the metabolism of diazepam, the metabolites were investigated instead^33^.

### Hydrolysis

Another reason for the limited NPS detection in this study may be the presence of glucuronide analogues formed during metabolism. A small study was designed to determine whether compounds are being missed due to their glucuronide analogues not being broken down without hydrolysis. Three out of the total seven wastewater samples collected were randomly selected for this experiment, and each sample was separated into 5 individual aliquots of 25 mL. Enzymatic β-Glucuronidase BG Turbo was added to each aliquot (550 μL), and the sample was placed in an incubator set at 55 °C. Each sample was spiked with an internal standard as a qualitative check that extraction was successful.

All samples were left in the incubator at 55 °C and removed at a specific time interval (0, 1 h, 2 h, 24 h, and 48 h). Once all samples had time to cool in the fridge, all samples underwent SPE, as mentioned above, and were then analysed on the LC-ToF–MS method. PeakView software was used to qualitatively process the results. Results of the hydrolysis experiment concluded that there is an increase in compound concentration through hydrolysis, meaning that there is a change in concentration due to the breakdown of conjugated compounds.

Using BG Turbo as the enzyme, it was evident that most compounds reached complete hydrolysis at 2 h, the only difference being that buprenorphine hydrolysis was instant. The hydrolysis was measured based on an increase in peak height for all compounds. In total, 6 compounds were investigated, all being the more common medicinal substances prescribed in a prison setting. These compounds are also known to have glucuronide analogues: morphine-glucuronide, codeine-glucuronide, buprenorphine-glucuronide, oxazepam-glucuronide, and nordiazepam-glucuronide.

**Figure 1:**
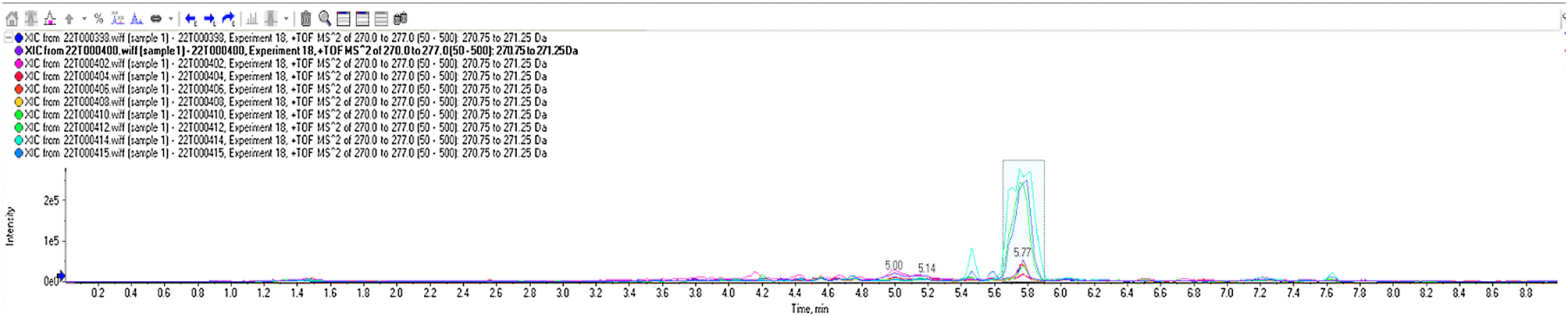
Image from PeakView illustrating the presence of Nordiazepam in each sample.

The results of one of the samples analysed (sample 6), showed a 200% increase in codeine between pre- and post-hydrolysis, indicating that the glucuronide analogue was preventing detection for codeine before 2-h hydrolysis. Before hydrolysis, there was no codeine detected in the sample. Nordiazepam was also showing between 173 and 172% increase in peak area from pre- to post-hydrolysis after 2 h. Figure 1 illustrates the peak for nordiazepam in every sample post-2-h hydrolysis.

It is well documented that these methods require hydrolysis when testing in urine but, Bijlsma et al. stated in their study that hydrolysis should not be a requirement with wastewater testing due to in-sewer deconjugation plus the potential removal from filtration and solid phase extraction^34–36^. But, based on this small study looking at glucuronide analogues of some of the more common medicinal substances, SPE does not optimally remove all analogues. Therefore, additional concentrations of analytes may be missed if hydrolysis is not carried out on wastewater samples if glucuronide analogues are present.

### Study limitations

A limitation of this study is the sample collection method. Grab/spot sampling has been successfully used in several wastewater studies^37,38^, but composite sampling with its ability to collect samples over a specified period (24 h) with intermittent collection points, can provide a more representative sample of an area. Spot collections provide a single snapshot of wastewater composition at a specific point in time. The grab sample may not capture the full range of target analyte concentrations within the wastewater, but it can provide a present or not-present outlook on drug usage within a particular area. Finally, grab sampling may not align with the peak drug usage periods which can lead to an underestimation of drug concentrations. Composite sampling has proven to provide advantages when required for wastewater collection and illustrating drug trends. One of the main disadvantages is the cost involved. Composite auto-samplers can be costly and without funding to purchase or hire an autosampler, grab sampling is a much more affordable approach. A study conducted in the USA discusses the testing of wastewater within a prison setting^21^. This study explored the use of composite testing where 24-h samples across 2 days were collected to observe all urine pulses potentially containing illicit substances. The study found a quantifiable concentration of methamphetamine every day but did not detect benzoylecgonine (the main metabolite of cocaine) above LOQ^21^. Another limitation of our study is the number of samples collected, a total of 7 samples were collected on the same day. This doesn’t allow a comparative study of the drug trend within the prison; the results only provide a present or not-present indication. The frequency of sampling was low due to access restrictions to the manholes where the samples were collected, this was because of the prison security arrangements that had to be put in place. This was also hindered by the staff shortages and backlog within the prison system to allow any further testing. Limitations of low sample frequency within a prison setting has also been observed in other similar studies^39^.

## Conclusion

Analysis of wastewater samples performed in the present study did not detect NPS within the Northern Ireland Prison. The study did provide evidence of the presence of cocaine use within the prison. Therefore, it is suggested that there is an illicit use of cocaine within the prisons when there is a strict no illicit/non-medicinal use policy. The negative finding for NPS could be caused by several factors, the NPS substances previously reported to be causing issues within prison settings may have evolved the target analytes are no longer relevant or the concentration of NPS is too low to be considered detectable due to the extensive dilution in water. Even though when a new NPS enters a population area, its uptake and popularity remain low in comparison to its illicit counterparts, the concentration within wastewater will remain very low. Even though no NPS were detected in this study, it is still evident that wastewater testing can be a vital tool for prison settings to utilise to explore any illicit substance abuse that is going undetected. It may be more beneficial for the prisons to adopt a more un-targeted, qualitative approach which in turn will reduce validation and development time but also be flexible to adapt to the ever-changing environment. Finally, if a prison setting requires a more comprehensive overview of the potential drug use among inmates, the adoption of a method that includes a 24-h autosampler will be beneficial and will provide the required data to achieve that objective.

### Supplementary Information

Supplementary Tables.

## Data Availability

The datasets used and/or analysed during the current study are available from the corresponding author upon reasonable request.

## Abbreviations

SWATH: Sequential window acquisition of all THeoretical fragmentation ion spectra
MeOH: Methanol
NPS: New psychoactive substances
EMCDDA: European monitoring centre for drugs and drug addiction
LC-ToF–MS: Liquid chromatography time-of-flight mass spectrometry
SWGTOX: Scientific working group for forensic toxicology
QC: Quality control
WWTP: Wastewater treatment plant
MDPV: Methylenedioxypyrovalerone
