# Supplementary material for: Wastewater analysis for new psychoactive substances and cocaine and cannabis in a Northern Ireland Prison
Source: Sci Rep. 2023 Oct 30;13:18634. doi: 10.1038/s41598-023-44453-4 (PMC10616220; doi:10.1038/s41598-023-44453-4)
Supplement: Supplementary file 1 — Supplementary Tables. [file 41598_2023_44453_MOESM1_ESM.docx]

Table S1 - Table illustrating the RT, SWATH window, fragment and precursor ion for each compound.

| Analyte | Retention Time | SWATH Window | Precursor Ion (m/z) | Fragment Ion (m/z) |
| --- | --- | --- | --- | --- |
| 25C-NBOMe | 5.04 | 28 | 336.13 | 121.06 |
| 25I-NBOMe | 5.25 | 44 | 428.07 | 121.06 |
| 2C-B | 4.54 | 16 | 260.01 | 242.98 |
| 2-OXO-LSD | 4.07 | 32 | 256.19 | 237.10 |
| 4-Methylethcathinone | 4.28 | 4 | 192.13 | 174.12 |
| 5F-AB-PINACA | 5.89 | 31 | 349.20 | 304.18 |
| 5F-APICA | 6.95 | 39 | 399.24 | 135.11 |
| 5F-APINACA | 6.97 | 39 | 400.23 | 135.11 |
| 5F-MDMB-PINACA | 6.49 | 33 | 364.20 | 318.20 |
| 5F-PB-22 | 5.75 | 14 | 250.12 | 162.05 |
| 5-MeO-DALT | 4.47 | 18 | 271.17 | 174.09 |
| AB-FUBINACA | 6.13 | 34 | 370.15 | 324.15 |
| AB-PINACA | 5.17 | 33 | 330.97 | 98.90 |
| AB-PINACA Metabolite | 6.39 | 28 | 361.19 | 344.16 |
| Alprazolam | 5.92 | 1 | 309.06 | 281.05 |
| AM2201 4-Hydroxypentyl | 6.62 | 35 | 376.17 | 155.05 |
| APICA 4-hydroxypentyl | 7.08 | 36 | 382.23 | 135.10 |
| APINACA 4-hydroxypentyl | 7.08 | 36 | 382.25 | 135.11 |
| APINACA 5-hydroxypentyl | 7.08 | 36 | 382.25 | 135.11 |
| Benzoylecgonine | 4.44 | 1 | 290.09 | 168.07 |
| Benzyl Piperazine | 1.54 | 1 | 177.05 | - |
| Etizolam | 6.00 | 30 | 342.07 | 314.00 |
| Fentanyl | 4.80 | 1 | 337.20 | 188.10 |
| JWH-018 Pentanoic Acid | 6.79 | 34 | 372.15 | 155.04 |
| LSD | 4.64 | 27 | 324.21 | 223.12 |
| MDMB-CHMICA | 5.89 | 34 | 371.20 | 240.14 |
| MDPV | 4.59 | 18 | 276.16 | 126.10 |
| Mephedrone | 4.16 | 2 | 178.11 | 160.10 |
| Methoxetamine | 4.48 | 14 | 248.16 | 203.10 |
| Methylone | 3.91 | 7 | 208.08 | 160.06 |
| Norfentanyl | 4.30 | 11 | 233.10 | 84.08 |
| PB-22 Carboxyindole | 4.58 | 11 | 232.13 | 188.14 |
| TFMPP | 4.59 | 11 | 231.09 | 188.05 |
| THC-COOH | 5.99 | 30 | 345.15 | 327.14 |
| UR-144 4-Hydroxypentyl | 6.63 | 27 | 328.22 | 125.09 |
| UR-144 5-Hydroxypentyl | 6.63 | 27 | 328.22 | 125.09 |
| UR-144 COOH | 6.60 | 30 | 343.30 | 240.23 |
| Alprazolam-D5 | 5.91 | 1 | 314.10 | 256.00 |
| JWH-018-D5 | 5.34 | 1 | 375.98 | 154.95 |
| Fentanyl-D5 | 4.83 | 1 | 341.00 | 187.91 |
| 25C-NBOMe-D3 | 5.09 | 1 | 338.95 | 123.87 |
| LSD-D3 | 4.67 | 1 | 327.04 | 281.65 |
| UR-144 5-hydroxypentyl-D5 | 6.62 | 1 | 333.00 | - |
| TFMPP-D4 | 4.60 | 1 | 234.85 | 189.66 |
| 25I-NBOMe-D3 | 5.32 | 1 | 430.90 | 123.84 |
| Benzoylecgonine-D3 | 4.60 | 1 | 292.99 | 170.82 |
| Benzylpiperazine-D7 | 3.81 | 1 | 184.29 | - |

Table S2 - Table illustrating the validation results.

| Analyte | Recovery (%) | Accuracy (%) | | | Precision (%) | | | | | |
| --- | --- | --- | --- | --- | --- | --- | --- | --- | --- | --- |
|  |  |  |  |  | Intra-day | | | Inter-day | | |
|  |  | Low | Mid | High | Low | Mid | High | Low | Mid | High |
| 25C-NBOMe | 36% | 100 | 86 | 84 | 18 | 12 | 8 | 19 | 16 | 12 |
| 25I-NBOMe | 25% | 89 | 81 | 91 | 16 | 12 | 14 | 19 | 13 | 17 |
| 2C-B | 24% | 100 | 93 | 87 | 20 | 18 | 17 | 28 | 26 | 20 |
| 2-OXO-LSD | 33% | 89 | 86 | 91 | 15 | 19 | 19 | 20 | 20 | 22 |
| 4-Methylethcathinone | 16% | 89 | 94 | 97 | 16 | 19 | 19 | 22 | 30 | 22 |
| 5F-AB-PINACA | 29% | 89 | 88 | 94 | 18 | 17 | 16 | 20 | 18 | 17 |
| 5F-APICA | 61% | 89 | 86 | 96 | 18 | 19 | 17 | 20 | 20 | 23 |
| 5F-APINACA | 59% | 100 | 81 | 77 | 16 | 18 | 15 | 20 | 19 | 25 |
| 5F-MDMB-PINACA | 59% | 89 | 97 | 99 | 18 | 17 | 14 | 19 | 18 | 16 |
| 5F-PB-22 | 65% | 100 | 83 | 86 | 19 | 20 | 17 | 20 | 25 | 20 |
| 5-MeO-DALT | 44% | 99 | 97 | 99 | 14 | 12 | 16 | 18 | 13 | 18 |
| AB-FUBINACA | 42% | 89 | 100 | 97 | 18 | 17 | 13 | 18 | 19 | 15 |
| AB-PINACA | 52% | 100 | 97 | 96 | 20 | 16 | 10 | 22 | 17 | 12 |
| AB-PINACA Metabolite | 80% | 100 | 88 | 97 | 18 | 18 | 12 | 19 | 24 | 13 |
| Alprazolam | 85% | 99 | 86 | 94 | 18 | 18 | 18 | 20 | 22 | 24 |
| AM2201 4-Hydroxypentyl | 57% | 100 | 83 | 87 | 17 | 19 | 16 | 24 | 22 | 18 |
| APICA 4-hydroxypentyl | 37% | 89 | 91 | 92 | 18 | 19 | 19 | 19 | 21 | 21 |
| APINACA 4-hydroxypentyl | 55% | 89 | 96 | 96 | 20 | 19 | 14 | 22 | 21 | 16 |
| APINACA 5-hydroxypentyl | 55% | 89 | 96 | 96 | 20 | 19 | 14 | 22 | 21 | 16 |
| Benzoylecgonine | 55% | 80 | 77 | 96 | 15 | 20 | 16 | 21 | 29 | 20 |
| Benzyl Piperazine | 30% | 100 | 97 | 89 | 20 | 16 | 19 | 25 | 21 | 23 |
| Etizolam | 42% | 100 | 91 | 96 | 17 | 11 | 15 | 18 | 14 | 17 |
| Fentanyl | 43% | 89 | 91 | 99 | 20 | 17 | 9 | 22 | 20 | 10 |
| JWH-018 Pentanoic Acid | 43% | 89 | 94 | 98 | 16 | 18 | 19 | 17 | 22 | 21 |
| LSD | 42% | 89 | 79 | 99 | 19 | 16 | 19 | 23 | 7 | 23 |
| MDMB-CHMICA | 51% | 100 | 94 | 95 | 18 | 17 | 18 | 19 | 19 | 20 |
| MDPV | 109% | 100 | 97 | 82 | 17 | 16 | 20 | 23 | 17 | 24 |
| Mephedrone | 24% | 100 | 97 | 98 | 16 | 18 | 17 | 19 | 16 | 24 |
| Methoxetamine | 30% | 89 | 88 | 89 | 13 | 19 | 16 | 14 | 19 | 21 |
| Methylone | 44% | 89 | 100 | 98 | 19 | 10 | 11 | 28 | 10 | 10 |
| Norfentanyl | 28% | 100 | 91 | 86 | 19 | 12 | 19 | 22 | 14 | 23 |
| PB-22 Carboxyindole | 30% | 100 | 100 | 99 | 17 | 18 | 18 | 18 | 20 | 22 |
| TFMPP | 24% | 89 | 100 | 100 | 16 | 17 | 10 | 17 | 21 | 12 |
| THC-COOH | 49% | 94 | 100 | 89 | 16 | 15 | 16 | 24 | 24 | 21 |
| UR-144 4-Hydroxypentyl | 54% | 89 | 91 | 95 | 20 | 11 | 16 | 24 | 13 | 21 |
| UR-144 5-Hydroxypentyl | 54% | 89 | 91 | 95 | 20 | 11 | 16 | 24 | 13 | 21 |
| UR-144 COOH | 66% | 94 | 100 | 89 | 18 | 7 | 15 | 21 | 25 | 19 |
